# Assessment of a Brazilian public policy intervention to address schistosomiasis in Pernambuco state: the SANAR program, 2011–2014

**DOI:** 10.1186/s12889-018-6102-5

**Published:** 2018-10-25

**Authors:** Luiz Augusto Facchini, Bruno Pereira Nunes, Eronildo Felisberto, José Alexandre Menezes da Silva, Jarbas Barbosa da Silva Junior, Elaine Tomasi

**Affiliations:** 1 Programa de Pós-Graduação em Epidemiologia, Universidade Federal de Pelotas, Pelotas, Brazil; 2 Programa de Pós-Graduação em Enfermagem, Universidade Federal de Pelotas, Rua Gomes Carneiro, 1, Campus ANGLO, Centro, Pelotas, RS CEP: 96010-610 Brazil; 3 Grupo de Estudos em Gestão e Avaliação em Saúde, Instituto de Medicina Integral Prof. Fernando Figueira, Recife, Pernambuco Brazil; 4 Instituto de Higiene e Medicina Tropical, Universidade NOVA de Lisboa, Lisboa, Portugal; 5 Pan American Health Organization (PAHO/AMRO/WHO), Washington - DC, USA

**Keywords:** Schistosomiasis, Collective treatment, Parasitic diseases, Prevalence, Health policy, Brazil

## Abstract

**Background:**

Brazil is an endemic country for schistosomiasis in the Latin American and Caribbean countries. Pernambuco is a higher-endemic Brazilian state among the 19 states reporting the disease in the country; schistosomiasis affects 102 (55%) of its 185 municipalities. Our objective was to evaluate the effectiveness of the treatment cycles of the SANAR Program (Plan to Reduce and Eliminate Neglected Diseases) in Pernambuco State in Northeast Brazil.

**Methods:**

A cross-sectional population-based study was conducted in 2014 via a household survey in 117 hyperendemic locations in the state of Pernambuco. We compared the schistosomiasis prevalence rates in hyperendemic locations, aggregated by geographical region, before and after the intervention. The dependent variable was a positive stool test result by the Kato-Katz method, and the main exposure variable was the number of treatment cycles (one/two). The covariables were the regions of the state and socioenvironmental, socioeconomic, demographic and behavioral characteristics.

**Results:**

In all, 12,969 individuals were interviewed, 8932 of whom had stool tests. Of these, 4969 (55.6%) underwent two cycles of collective treatment. Changes in the environmental conditions since 2011 were minimal. Comparison before (2011) and after (2014) treatment showed an average schistosomiasis prevalence of 18.6%, decreasing to 4.1% and 2.0% in locations with one and two treatment cycles, respectively. In 2014, the highest schistosomiasis prevalence was found in the forest area (2.8%), while the lowest was found in the northern region (1.2%) of the state. The adjusted analysis showed a lower occurrence of schistosomiasis in individuals living in areas with two treatment cycles than in individuals from areas with just one cycle (PR 0.65, 95% CI: 0.47–0.89).

**Conclusions:**

The political decision made in Pernambuco to implement the SANAR Program in 2011 greatly impacted the burden of schistosomiasis. This program was effective in reducing the occurrence of schistosomiasis in hyperendemic areas in Pernambuco, with a stronger response in areas with two cycles of collective treatment.

## Background

Schistosomiasis is an infectious disease caused by digenetic trematodes of the *Schistosoma* genus. In 2015, schistosomiasis affected approximately 250 million people worldwide [1]. The disease is transmitted by *Schistosoma mansoni* in the Region of the Americas, and Brazil and Venezuela present the highest endemicity [2, 3]. Between 2.5 and 6 million people are currently estimated to be infected with *S. mansoni* [4], and 700–800 deaths are reported annually in 19 Brazilian endemic states [5, 6]. A recent systematic review and meta-analysis reported that the prevalence of *S. mansoni* infection in Brazil varied widely, from 0·1 to 73·1%, based on the Kato-Katz technique [7].

Pernambuco is one of the states where schistosomiasis is endemic; in Pernambuco, schistosomiasis affects 102 (55%) of the 185 municipalities, with regional differences [8], and between 10 and 50% of test results are positive [9, 10]. The death and hospitalization rates related to schistosomiasis are significantly higher in Pernambuco than in other Brazilian states [11]. The low socioeconomic level and scarcity of knowledge about the problem of schistosomiasis increase the transmission of the disease, particularly among children and adults, because of participation in recreational and occupational activities [12].

In addition to involving improved sanitation conditions and treated drinking water availability [10, 13], the control of schistosomiasis involves early diagnosis, timely treatment, surveillance and educational actions [14]. In endemic areas with positivity rates equal to or greater than 10%, schistosomiasis control using praziquantel is recommended by means of large-scale treatment of the population. The strategy of treating all individuals greater than two years of age [15] is associated with a decrease in the worldwide schistosomiasis occurrence [15, 16].

A single dose of medication is considered an effective collective treatment and is a safe and low-cost strategy for controlling all forms of schistosomiasis [17]. In 2014, more than 60 million people were treated for schistosomiasis worldwide [18].

In keeping with World Health Organization (WHO) proposals, in 2011, Brazil launched its Integrated Strategic Action Plan for the Elimination of Neglected Diseases [4]. In cooperation with state- and municipal-level health service managers, the plan proposes the active searching for and timely treatment of cases, considering, when indicated, collective treatment interventions in locations with positive test results in more than 25% of the population [14]. In 2011, the Pernambuco State Health Department launched the SANAR Program to reduce and/or eliminate the following neglected diseases: schistosomiasis, tuberculosis, leprosy, Chagas disease, leishmaniasis, filariasis, geohelminthiases and trachoma. SANAR is the first initiative of the integrated plan at the state level to develop a specific program for combating schistosomiasis by including collective treatment. The main integrated SANAR actions were community education and environmental assessment in hyperendemic areas for schistosomiasis [10, 19], supplemented by household collective treatment. At the management level, the program established guidelines, goals and indicators that were routinely monitored as part of the priority actions of the Pernambuco State Government [20, 21]; these parameters were associated with the training of health workers, particularly primary health care teams, community health workers, and personnel from endemic control, health surveillance, information systems, and laboratory organizations [22].

In contrast with the national reference and in line with the WHO recommendations [15], the political decision made in Pernambuco was to maximize the effectiveness of intervention; to this end, the Pernambuco State Health Department adopted collective treatment for areas where the schistosomiasis prevalence in the population was greater than 10%. This intervention was integrated with selective treatment involving family health teams that was made available also in endemic areas with prevalence rates below 10% [23].

Given the social relevance of the SANAR Program to reduce and eliminate schistosomiasis as a neglected disease, this article aims to evaluate the effectiveness of this program according to the collective treatment cycles. To contextualize the relevance of the findings of the effectiveness study, we previously assessed the trends for disease positivity, treatment, hospitalization and mortality during the period 2010–2014 both in Pernambuco and in Brazil.

## Methods

### Schistosomiasis trends in Pernambuco and in Brazil

The trend study was conducted based on secondary data routinely collected in Brazil and obtained from health information systems. The data on the positivity of tests performed to confirm schistosomiasis and on the treatment of confirmed cases were provided by the Information System for Notifiable Diseases (SINAN), the data on hospitalization owing to schistosomiasis were obtained from the Hospital Information System (SIH-SUS), and the data on deaths due to schistosomiasis (B65 − ICD-10) were obtained from the Mortality Information System (SIM). The data in the three databases were both for the state of Pernambuco and for the country of Brazil for the same period: 2010–2014.

### SANAR program: selection of locations for collective treatment interventions

Based on the Schistosomiasis Control Program Information System (SIS-PCE/SES-PE), a total of 119 hyperendemic locations in rural and urban areas of 30 priority municipalities were identified in the state of Pernambuco for the years 2010 and 2011. The municipalities that had a mean prevalence of 10% or more between 2005 and 2010 were included in the program [23]. In each endemic municipality, all locations with a schistosomiasis prevalence greater than or equal to 10% were included [24].

In the 119 locations selected, three cycles of collective treatment using chemotherapy were planned to take place, with a minimum planned coverage of 80% of the target population. In 2012 and 2013, 61 (52.1%) locations received one treatment cycle and 56 (47.9%) received two cycles. Approximately 70% of the locations had a mean coverage greater than 70% (lower: southern = 67%; higher: metropolitan zone = 79%), with a median coverage of 79%.

Praziquantel manufactured by Farmanguinhos was used for treatment at a dose of 50 mg *per* kilo of body weight (mg/kg) for people over 15 years old and 60 mg/kg for children between two and 15 years old. The recommended dosage is different because of differences in the metabolism of adults and children. Praziquantel was provided in tablet format and administered orally in a single dose. Adverse effects are slight, and there is no evidence that praziquantel produces serious toxic lesions of the liver or other organs [2, 15, 24].

### Effectiveness evaluation survey

In September and October 2014, a cross-sectional population-based study was conducted via a household survey to evaluate the performance of the SANAR program in relation to schistosomiasis in 117 hyperendemic locations. The survey compared the schistosomiasis prevalence rates before and after the intervention in hyperendemic locations aggregated by geographical region. The sampling process selected a representative sample in all regions with endemic areas stratified by the number of collective treatments performed. A starting point was chosen in each locality, and from this starting point, a systematic sample of households was included. The quota was proportional to the size of the locality. The higher/lower the population of the locality, the higher/lower was the number of households selected. The number itself was based on this proportionality and on the final intended sample size. The final sample population was 11,842. All individuals in each household aged over two years old were included. Pregnant women were excluded.

The data were collected by health surveillance personnel from the State Health Department of Pernambuco using handheld computers following one week of training. If an eligible family member was absent, the data for that individual were obtained from a key informant. At the same time as the data collection, marked plastic containers were provided for the collection of stool samples from the household the next day. The samples were kept on ice in Styrofoam coolers. On the third day, the coolers were delivered to the laboratory, where they were stored in refrigerators until the preparation and reading of microscope slides. Laboratory analysis took place between the first week of September and the end of October 2014. Before being read under a microscope, the slides were prepared by mid-level professional staff at the schistosomiasis laboratory of the municipality of Jaboatão dos Guararapes-PE. The Kato-Katz method was used [25]. This predominant method for detecting *S. mansoni* eggs is relatively straightforward, inexpensive and highly specific [26–29]. Valid stool samples were those for which the stool quantity was sufficient to allow the sample to be processed via the Kato-Katz method. Some of the collection containers delivered had no stool, only a small amount of stool, or stool of a liquid or pasty consistency, which prevented processing for slide preparation. The test results were read by biologists from the State Laboratory of Public Health working for the schistosomiasis control program. The results were recorded on the epidemiological surveillance field form and sent for processing at the State Health Department. After input, copies of the forms were sent to the municipalities to be entered into the SISPCE and to allow the treatment of positive cases.

The dependent variable was a positive stool test result for Manson’s schistosomiasis, and the main exposure variable was the number of treatment cycles (one/two). The other exposure variables were the following: “region of the state” (northern (*Agreste Setentrional*)/metropolitan/southern (*Agreste Meridional*)/forest area (*Zona da Mata*)); “zone of residence”; the presence of open drainage ditches/sewers; “positive house flooding status”; “economic status” according to the Brazilian Association of Studies and Research (B and C/D/E); “sex” (female/male); “age” (< 5/5–14 years/15–44 years/45 years or over); “skin color or race” (Caucasian, Afro-Brazilian, Asian, Mixed/Pardo Brazilian, Amerindian); “cohabitation status”; “contact with river water”; “contact with water from open drainage ditches/sewers”; “walking barefoot in the street”; and “schistosomiasis occurrence at some time in life”.

The raw and adjusted analyses were performed using Poisson regression with robust variance adjustment [30]. The adjusted analysis aimed to evaluate the effect of the number of treatment cycles on the occurrence of schistosomiasis. To this end, a hierarchical analysis model was used with the aim of determining which variables influenced the association. The adjusted analyses comprised four levels (adjustment 1: “region of the state” and “zone of residence”; adjustment 2: “positive house flooding status” and “economic status”; adjustment 3: “sex”, “age” and “skin color”; adjustment 4: “contact with river water” and “schistosomiasis occurrence at some time in life”).

## Results

### Schistosomiasis trends in Pernambuco and in Brazil

Almost 100,000 and 1,382,785 tests to assess for schistosomiasis positivity were performed in Pernambuco and Brazil, respectively, in 2010; 256,809 and 814,905 tests were performed in Pernambuco and Brazil, respectively, in 2014. Schistosomiasis positivity was 8.31% in 2010 and 3.39% in 2014 in the state of Pernambuco (during these same years, the positivity decreased from 5.02 to 4.07% in Brazil). In Pernambuco, the proportion of individuals with positive test results who were treated increased, while in Brazil, this proportion decreased. The rate of hospitalization from schistosomiasis (per 100,000 inhabitants) decreased in both Pernambuco and Brazil. The schistosomiasis mortality rate in Pernambuco decreased from 2.23 (per 100,000 inhabitants) in 2010 to 1.51 in 2014. The total mortality rate for Brazil was 0.27 in 2010 and 0.24 in 2014 (Please see Table 1).

**Table 1:** Indicators of schistosomiasis positivity, hospitalization and mortality in Pernambuco and Brazil, 2010–2014

Indicators	Pernambuco						Brazil					
	2010	2011	2012	2013	2014	2014/2010	2010	2011	2012	2013	2014	2014/2010
Diagnosis and treatment												
Tests performed^b^	98,561	103,880	94,384	120,171	256,809	2.61	1,382,785	1,267,247	878,751	795,174	814,905	0.59
Positive result	8186	7623	5336	6509	8713	–	69,436	59,946	38,685	36,994	33,193	–
Positivity (%)	8.31	7.34	5.65	5.42	3.39	0.41	5.02	4.73	4.4	4.65	4.07	0.81
Treated	6500	6027	4300	5197	7104	–	61,820	52,238	29,506	28,272	26,042	–
Treatment (%)	79.4	79.1	80.6	79.8	81.5	1.03	89.0	87.1	76.3	76.4	78.5	0.88
Hospitalizations												
Hospitalizations due to schistosomiasis	78	62	53	33	41	–	301	240	218	163	175	–
Hospitalization rate (per 100,000 inhab.)	0.89	0.70	0.59	0.36	0.44	0.50	0.16	0.12	0.11	0.08	0.09	0.55
Mortality												
Schistosomiasis deaths (B65)	196	174	158	141	140	–	514	546	488	468	480	–
Total deaths	54,570	57,219	57,132	58,209	57,823	–	1,136,947	1,170,498	1,181,166	1,210,474	1,227,039	–
Proportional schistosomiasis mortality (%)	0.36	0.30	0.28	0.24	0.24	0.67	0.05	0.05	0.04	0.04	0.04	0.87
Schistosomiasis mortality rate (per 100,000 inhab.)^a^	2.23	1.96	1.77	1.53	1.51	0.68	0.27	0.28	0.25	0.23	0.24	0.88

^a^Total population - Pernambuco, 2010: 8796032, 2011: 8864906, 2012: 8931028, 2013: 9208550, 2014: 9277727; Brazil: 2010: 190747855, 2011: 192379287, 2012: 193946886, 2013: 201032714, 2014: 202768562

^b^Population accessed - Pernambuco, 2010: 137363, 2011: 140503, 2012: 133990, 2013: -, 2014: 358954; Brazil, 2010: 1849983, 2011: 1709702, 2012: 1205789, 2013: 1111304, 2014: 1127632

### Effectiveness evaluation survey

A total of 12,969 individuals were interviewed; valid stool samples were collected for evaluation from 8932 (68.9%) of these. Among the infected study subjects (*n* = 197), the mean number of eggs per slide was 7.9 (189.3 per gram), the median was 2 per slide (48 per gram), the interquartile interval per slide was 1–6 (24–144 per gram), and 52.3% of the subjects presented 1 or 2 eggs per slide by examination (more than 48 eggs per gram). A total of 4969 (55.6%) of the individuals underwent two treatment cycles. The forest area (*Zona da Mata*) region had the highest proportion of interviewees (31.7%). Half of the interviewed individuals lived in the urban zone. A total of 41.2% of the individuals lived in locations with open drainage ditches/sewers that had been corrected in fewer than 3% of these locations. Water usually flooded the houses of 11.2% of the individuals, while 27.5% reported that there had never been flooding where they lived. Only 3.1% stated that the flooding situation had improved since 2011. The majority of the interviewees were of economic status D (58.8%). Half of the sample was female, and half was aged 15–44. The most commonly reported skin color was brown (62.3%), and 45.9% reported having a partner. Almost one-third reported having current contact with river water (28.7%) and with water from open drainage ditches/sewers (33.4%). Less than 15.0% stated that they always walked barefoot in the street, and 61.9% stated that they never did this. A diagnosis of schistosomiasis at some time in life was reported by 17.1% of the interviewees (Please see Table 2).

**Table 2:** Sample characteristics, schistosomiasis prevalence and raw analysis according to independent variables. Pernambuco, Brazil, 2014

Variables	n	%	% Schistosomiasis	Raw analysis			*p*-value
				PR	95% CI		
Number of treatment cycles							0.011
1	3963	44.4	2.7	1.00			
2	4969	55.6	1.9	0.70	0.53	0.92	
Region							0.001
Northern (*Agreste Setentrional*)	2415	27.0	1.2	1.00			
Metropolitan	2400	26.9	2.5	2.12	1.37	3.28	
Southern (*Agreste Meridional*)	1290	14.4	2.1	1.74	1.04	2.93	
Forest area (*Zona da mata*)	2827	31.7	2.8	2.36	1.55	3.59	
Zone of residence							0.105
Urban	4878	55.0	2.4	1.00			
Rural	3992	45.0	1.9	0.79	0.60	1.05	
Open drainage ditch/sewer							0.999
No	5168	58.9	2.2	1.00			
Yes	3614	41.2	2.2	1.00	0.75	1.33	
Was there an open drainage ditch/sewer that was corrected after 2011?							0.860
No	8535	97.2	2.2	1.00			
Yes	247	2.8	2.0	0.92	0.38	2.23	
Does water usually flood houses?							0.039
Yes	981	11.2	2.8	1.00			
No	5380	61.3	2.4	0.86	0.57	1.30	
There has never been a flood	2417	27.5	1.6	0.57	0.35	0.93	
Was there flooding, but the situation improved after 2011?							0.114
No	8505	96.9	2.3	1.00			
Yes	273	3.1	0.7	0.33	0.08	1.31	
Economic status							0.001
B and C	2739	33.5	2.3	1.00			
D	4801	58.8	2.0	0.86	0.63	1.19	
E	626	7.7	4.3	1.91	1.22	2.97	
Sex							0.020
Female	4860	54.4	1.9	1.00			
Male	4072	45.6	2.6	1.39	1.05	1.83	
Age (full years)							0.002
< 5	738	5.7	0.6	1.00			
5–14	2746	21.2	1.6	2.42	0.74	7.91	
15–44	6335	48.9	2.8	4.35	1.39	13.6	
45 or over	3145	24.2	2.0	3.11	0.97	9.96	
Skin color							0.021
White	2443	27.5	1.5	1.00			
Black	848	9.5	3.2	2.10	1.29	3.43	
Brown	5546	62.3	2.4	1.57	1.09	2.26	
Yellow/indigenous	63	0.7	1.6	1.05	0.15	7.52	
Lives with a partner							0.421
No	4832	54.1	2.1	1.00			
Yes	4100	45.9	2.3	1.12	0.85	1.48	
Has contact with water from rivers							< 0.001
No, never	2263	25.6	1.1	1.00			
Yes, in the past but not currently	4046	45.7	2.6	2.30	1.49	3.56	
Yes, currently	2537	28.7	2.7	2.46	1.56	3.88	
Has contact with water from open drainage ditches/sewers							0.627
No, never	4180	47.3	2.2	1.00			
Yes, in the past but not currently	1705	19.3	2.5	1.11	0.77	1.59	
Yes, currently	2950	33.4	2.0	0.91	0.66	1.26	
Walks barefoot in the street							0.999
Never	5509	61.9	2.2	1.00			
Sometimes	1714	19.3	2.2	1.01	0.70	1.45	
Nearly always	526	5.9	2.3	1.04	0.58	1.87	
Always	1154	13.0	2.2	0.99	0.64	1.51	
Has had schistosomiasis at some time in life							0.193
No	7107	82.9	2.1	1.00			
Yes	1467	17.1	2.7	1.26	0.89	1.78	

The highest schistosomiasis prevalence among all four regions was found in the forest area (*Zona da Mata*) (2.8%, *p* = 0.001). The schistosomiasis prevalence in areas with two treatment cycles was 30% lower than that in areas with just one treatment cycle (*p* = 0.011). Individuals who lived in homes that flooded, were of economic status E, were male, were aged 15–44, stated that their skin color was black or brown, and had current and past contact with river water presented a higher prevalence of schistosomiasis. Of the interviewees, 17.1% stated that they had been diagnosed with schistosomiasis at some time in their lives (Please see Table 2). Those who had had the disease stated that the main symptoms were abdominal pain (47%) and feeling sick (35%).

The schistosomiasis prevalence in areas with two treatment cycles was approximately 35% lower than that in locations with just one treatment cycle, even after the adjustments (Please see Table 3).

**Table 3:** Raw and adjusted analyses of the association between schistosomiasis and medication treatment cycle. Pernambuco, Brazil, 2014

	Treatment cycle^a^		*p*-value
	PR	95% CI	
Raw analysis	0.70	0.53–0.92	0.011
Adjustment 1	0.64	0.47–0.87	0.005
Adjustment 2	0.58	0.42–0.81	0.001
Adjustment 3	0.61	0.44–0.83	0.002
Adjustment 4	0.65	0.47–0.89	0.008

^a^Reference group: 1st treatment cycle; Exposed group: 2nd treatment cycle

Adjustment 1: region of the state and zone of residence

Adjustment 2: Adjustment 1 + positive house flooding status and economic status (ABEP)

Adjustment 3: Adjustment 2 + sex, age and skin color

Adjustment 4: Adjustment 3 + contact with river water + schistosomiasis at some time in life

*PR* prevalence ratio. *95% CI* 95% confidence interval. *p*-value: Wald’s heterogeneity test

**Fig. 1:**
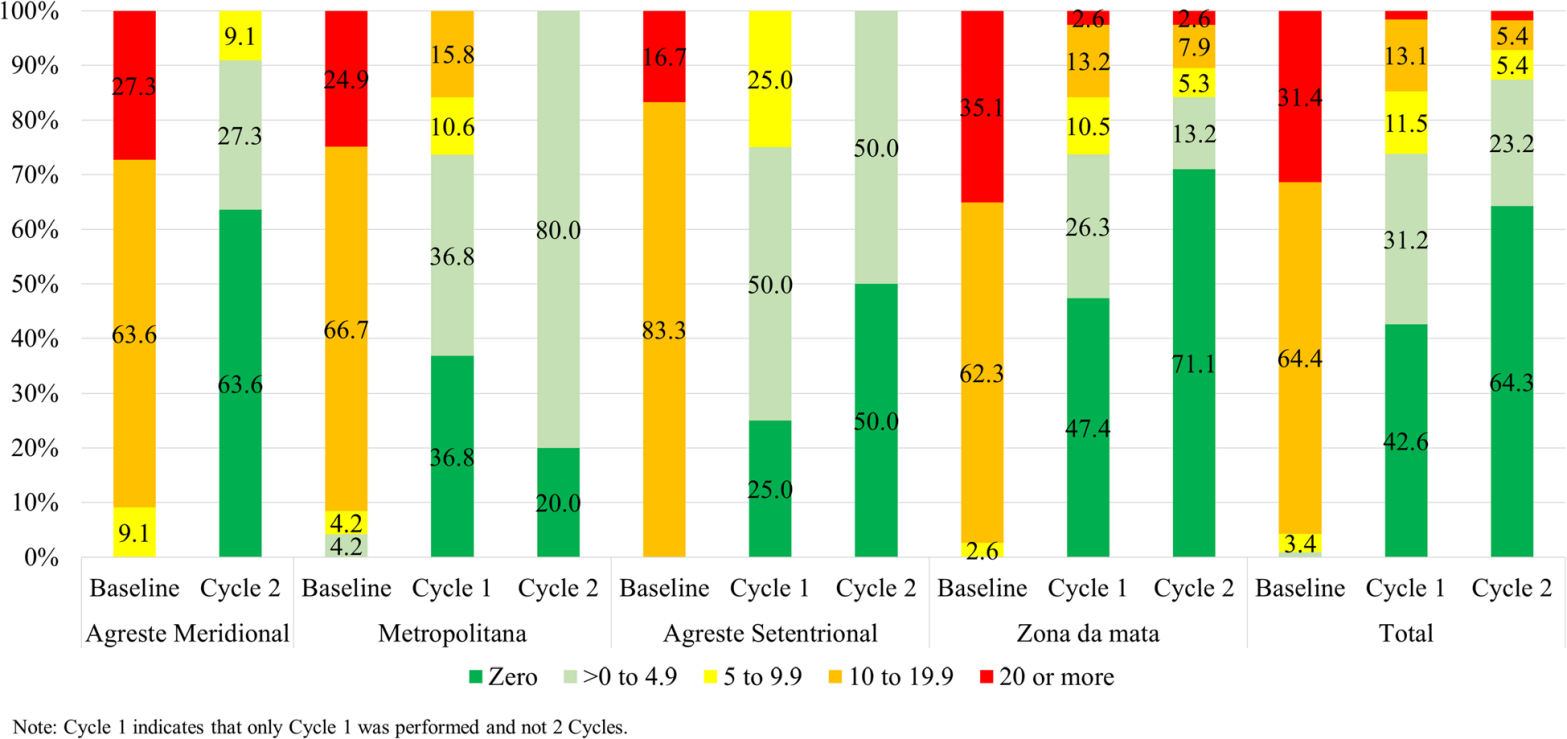
Schistosomiasis prevalence intervals by treatment cycle and region. Pernambuco, Brazil, 2011-2014

**Fig. 2:**
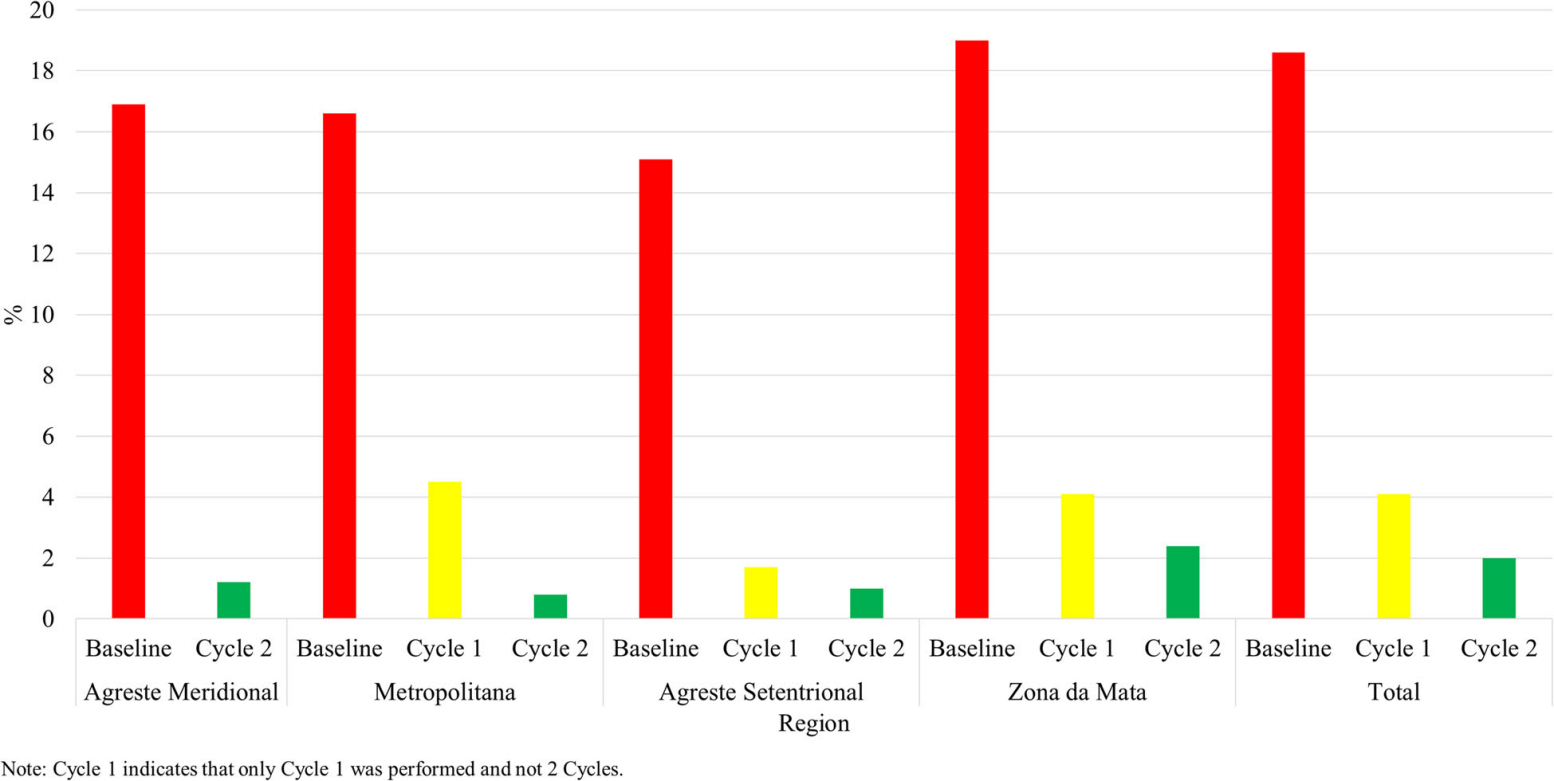
Schistosomiasis prevalence by treatment cycle and region. Pernambuco, Brazil, 2011-2014

Generally, before treatment, 64.4% of the locations had schistosomiasis prevalence rates of between 10 and 19.9%, whereas 31.4% had prevalence rates of 20% or greater. In areas with one and two treatment cycles, this proportion decreased to 13.1% and 5.4%, respectively. More robust reductions were found in the southern and northern regions than in the other regions. After two cycles of collective treatment, 64.3% of the locations studied had a schistosomiasis prevalence equal to zero (Please see Fig. 1). The average schistosomiasis prevalence was 18.6% at baseline, decreasing to 4.1% and 2.0% in areas with one and two treatment cycles, respectively (Please see Fig. 2).

## Discussion

SANAR provide a tested example for Brazil to compose its response to Sustainable Development Goals (SDGs). For the first time, the global agenda faces the explicit challenge to end epidemics of neglected tropical diseases by 2030, among other targets related to Goal 3: ensure healthy lives and promote well-being for all at all ages [31].

Repeated treatment during childhood and adolescence persists into adulthood, reducing the chronic evolution of schistosomiasis in hyperendemic areas, thus increasing the quality of life. Accurate monitoring in the next decade is essential to control not only the morbidity but also the mortality and severity of the disease, strengthening the effect of large-scale preventive chemotherapy regimens [32].

SANAR has provided new protocols and strategies for health surveillance actions for schistosomiasis in the state of Pernambuco, emphasizing the relevance of neglected tropical diseases in the political agenda of the state government. The overall cost of specific interventions for schistosomiasis to the state government, including the actions of the SANAR Program, was approximately US$ 308,081 in the period from 2011 to 2014, or about US$ 6000 per month; these expenses included inputs, printed matter, equipment, personnel wages, consultancies, training, and fuel. Through the Integrated Strategic Action Plan for the Elimination of Neglected Diseases, the Ministry of Health freely delivers praziquantel to the municipalities to guarantee collective treatment in the priority areas [4]. Unfortunately, the country’s current political scenario of funding restrictions on health policies for the next 20 years is threatening the program’s sustainability [33].

In keeping with the global trend of reduced schistosomiasis occurrence, which was estimated to be 23.5% between 2005 and 2015 [1], the detection of positive cases was found to have decreased in Brazil as a whole and in Brazilian states with high endemicity such as Pernambuco [11]. When the secondary data were used to analyze the timespan from 2010 to 2014 in Pernambuco, the reduction was significantly greater than that in Brazil and in the states of Sergipe and Alagoas (two states neighboring Pernambuco with similar schistosomiasis endemicity) [5]. The success achieved in controlling schistosomiasis in Pernambuco, compared to that achieved in the national and regional context, can essentially be attributed to the SANAR Program, which has not yet been implemented in the country’s other states. The possibility of comparing Pernambuco’s results with those achieved in Brazil as a whole and in neighboring states is a natural experiment [34]. An important part of the achievement attains plausibility via the adoption of collective treatment in locations with a prevalence greater than 10%, as recommended by the WHO [15], instead of the implementation of the Brazilian Ministry of Health’s recommendation, which is based on a prevalence of greater than 25% as the cutoff point for collective interventions [4].

When the primary data related to the biological material collected was evaluated, a beneficial effect of collective treatment was found in the reduction of positive schistosomiasis cases in hyperendemic locations. After two treatment cycles had been performed, approximately nine of every ten locations had a schistosomiasis prevalence of < 10%, the limit defined by the WHO as a low community risk for the propagation of the disease [35].

The finding that a higher number of treatment cycles had a protective effective on schistosomiasis occurrence in priority locations in Pernambuco reinforces the ability to control the disease in hyperendemic locations by means of collective treatment integrated with primary health care service actions, particularly the Family Health Strategy [36, 37]. Although socioenvironmental changes are one of the most important determinants of schistosomiasis [13, 37], few such changes occurred during the period of the SANAR Program activities [21], thus reducing the possibility that an environmental effect explains the reduction in schistosomiasis prevalence. In terms of health impacts and cost-effectiveness, few interventions rival collective treatment of neglected diseases integrated with primary health care. In addition to its relevance for people with schistosomiasis, this treatment approach has been increasingly recognized for its beneficial effects on strengthening health systems and on economic development [38].

Despite the relevance of the finding regarding the effectiveness of the intervention spearheaded by the SANAR Program, the evidence also shows that collective treatment alone is not sufficient to eliminate the disease. The SANAR Program promoted the integration of collective actions with Pernambuco’s primary health network, particularly with multidisciplinary family health teams. As the main expression of the Integrated Strategic Action Plan for the Elimination of Neglected Diseases in the state of Pernambuco, the SANAR Program intervention had a marked effect on increasing the number of people examined and treated between 2011 and 2014 [4].

The evidence from this study points to the relevance of continuing to monitor hyperendemic regions along with strengthening vector control and health services, the contribution of which to the effectiveness of the SANAR Program deserves to be evaluated in depth. Nevertheless, it is essential to reaffirm the key nature of implementing substantial environmental improvements in order to permanently eliminate and/or control schistosomiasis, thus valuing the quality of life and dignity of the population and, even more importantly, addressing the social determinants of the problem [10, 13, 22, 37].

Some authors [12] suggest that environmental changes can be more feasible if funding for programs to address neglected diseases includes allocating part of the resources to health activities, including the purchase and distribution of medication, and allocating another part of the resources to investments in extending treated drinking water networks and adequate sanitation and in improving living conditions [37].

The relevance of the socioenvironmental determinants of the problem is reinforced by noting that the overall success of the SANAR Program did not overcome the geographical context. The findings indicate that coastal regions and regions near watercourses (forest area/*Zona da Mata*), continue to have the highest prevalence rates and have higher proportions of locations with prevalence rates above 10% than do other regions [8, 12].

Although the study included poor and vulnerable locations, socioeconomic differences were strongly associated with the occurrence of schistosomiasis, thus highlighting the relevance of accounting for the heterogeneity of the population, even in locations considered to be contextually similar.

Some limitations of this study need to be considered. First, it was not possible to select the same individuals before and after collective treatment, which may have diluted the effect of treatment. However, given the large differences found, we do not believe that this limitation influenced the results in a significant manner. Second, the use of the Kato-Katz method to diagnose schistosomiasis in areas of low endemicity has been questioned. Some authors suggest that more sensitive techniques, such as ELISA, should be used [26]. However, the prolonged presence of reactive IgM after treatment and the death of the worms is a critical problem in evaluating the effectiveness of an intervention. We suggest that more sensitive techniques be used in future studies in high endemicity areas that become low prevalence areas following collective treatment in order to monitor changes via the complementary use of more sensitive techniques such as serial stool tests. Another limitation is that schistosomiasis surveys in the untreated locations, which could be used as controls, were not conducted. Furthermore, information on the snail vectors and their infection status in the study area is lacking.

The SANAR Program demonstrated effectiveness in controlling schistosomiasis in areas with high endemicity, so its implementation in other regions of Brazil and across the world can be recommended via adopting collective treatment in areas with a positive test prevalence in 10% or more of the population. The integrated action of the SANAR Program with the Family Health Strategy augments the interaction of the effects arising from collective treatment, prevention, surveillance and selective treatment. Despite the short time during which the Program has been implemented, its benefits for the population go beyond the significant reduction in the number of individuals with positive schistosomiasis test results, extending to a reduction in hospitalization and mortality from this disease.

## Conclusions

The political decision made in Pernambuco to implement the SANAR Program in 2011 greatly impacted the burden of schistosomiasis. The program promoted an effective reduction in the occurrence of schistosomiasis in hyperendemic areas in Pernambuco. The response was stronger in areas with two cycles of collective treatment than in one-cycle areas. The SANAR Program should be evaluated by other Brazilian states and even globally as a successful strategy for facing the challenge of neglected diseases.

## Abbreviations

95% CI: 95% confidence interval
PE: Pernambuco State
PR: Prevalence ratio
SANAR Program: Plan to Reduce and Eliminate Neglected Diseases
SDG: Sustainable development goals
SIH-SUS: Hospital Information System
SIM: Mortality Information System
SINAN: Information System for Notifiable Diseases
SISPCE: Schistosomiasis Control Program Information System
SISPCE/SES-PE: Schistosomiasis Control Program Information System of the Pernambuco State Government
WHO: World Health Organization
